# Flow uniformity data on 3D printed flow distributors

**DOI:** 10.1016/j.dib.2019.103799

**Published:** 2019-02-28

**Authors:** Mariana Garcia Mendonça Lopes, Harrson Silva Santana, Vinícius Felix Andolphato, João Lameu Silva, Osvaldir Pereira Taranto

**Affiliations:** a University of Campinas, School of Chemical Engineering, 13083-852 Campinas, SP, Brazil; b Federal Institute of Education, Science and Technology of South of Minas Gerais – IFSULDEMINAS, 37560-260 Pouso Alegre, MG, Brazil

**Keywords:** Distributors, 3D printed, Flow, Micro-chemical plant, Non-uniformity flow coefficient

## Abstract

Micro-chemical plants are chemical plants that use micrometrics structures by performing a numbering-up of microdevices. The flow distributor is responsible for the uniform distribution through these microdevices. Inadequate designs reduce the plant performance. Thus, 3D printing is a good alternative to allow fast and economic development for design tests. The present research applied 3D printing to manufacture flow distributors and evaluated the flow uniformity from experimental tests. This data article presents values of the non-uniformity flow coefficient (Ф) of three distinct distributors: the rectangular distributor (RD), the conical distributors with obstacle (CDO) and the conical distributor without obstacle (CD). The distributors were tested in water flow and at low flow rates, it was observed a flow maldistribution, related to the presence of air bubbles. For high flow rates the Ф reduced to values below 1%. The results presented here were used to validate the numerical simulations of flow distributors for numbering-up of biodiesel synthesis in micro and millidevices, “CFD analysis of flow distributor designs for numbering-up of biodiesel synthesis” [1].

**Table:** Specifications table

Subject area	*Manufacturing Engineering*
More specific subject area	*3D printing*
Type of data	*Tables, figures and graphs*
How data was acquired	*Flow uniformity:* using graduate test tubes and chronometers *Printing data: Simplify3D software*
Data format	*Raw and analyzed data*
Experimental factors	*3D printing (procedure in this article)*
Experimental features	*3D printer was used to manufacture flow distributors and the flow uniformity was evaluated from water flow tests using graduate test tubes and chronometers*
Data source location	*Campinas, Brazil, (22°54′25.57″S – 47°3′47.66″W)*
Data accessibility	*Data is included in this article*
Related research article	*CFD analysis of flow distributor designs for numbering-up of biodiesel synthesis.* Chemical Engineering Research and Design, 138, (2018) 458–471 [1].

**Table:** 

**Value of the data** • The experimental data was used for numerical simulation validation and verification procedure. • The data presents the uniformity flow of distributor designs in water flow experiments. • The description of material and methods of 3D printing technique could be applied to the manufacturing of flow distributors.

## 1 Data

The non-uniformity flow coefficient data of RD, CDO and CD distributors are presented in Table 1. Part of the results were presented in Refs. [2], [3] and the discussion of them contributed to the presentation in the present form. The data of CDO13, CDO52 and CD26 were present in article *CFD analysis of flow distributor designs for numbering-up of biodiesel synthesis* [1].

**Table 1:** Data of non-uniformity flow for distributors designs in water flow experiments.

Distributors (Ф)	Flow Rate (mL min^−1^)					
	100	200	240	300	340	400
RD13	34.22	17.48	11.80	4.46	5.40	2.04
RD26	4.78	2.99	3.75	2.50	3.28	0.73
RD52	13.96	5.94	6.64	4.37	2.01	3.32
CDO26	2.13	1.92	1.09	1.78	0.94	1.39
CD13	2.30	0.69	0.93	0.63	1.16	2.08
CD52	4.29	0.65	0.96	1.14	1.40	5.07

### 1.1 Averaged non-uniformity flow coefficients for the different distributors

Table 2 shows the average Ф values for all flow rates tested, disregarding the total flow rate of 100 mL min^−1^.

**Table 2:** Averaged non-uniformity flow coefficients for the 9 distributors.

Distributor	Averaged Ф (%)
CDO13	0.839 ± 0.418
CDO52	0.858 ± 0.308
CD26	0.970 ± 0.113
CD13	1.100 ± 0.527
CDO26	1.426 ± 0.380
CD52	1.844 ± 1.632
RD26	2.650 ± 1.043
RD52	4.458 ± 1.686
RD13	8.236 ± 5.636

For the simulation analysis the CDO52, CDO13 and CD26 designs were evaluated (CFD analysis of flow distributor designs for numbering-up of biodiesel synthesis [1]).

## 2 Experimental design, materials and methods

### 2.1 3D printing and flow distributor design

**Fig. 1:**
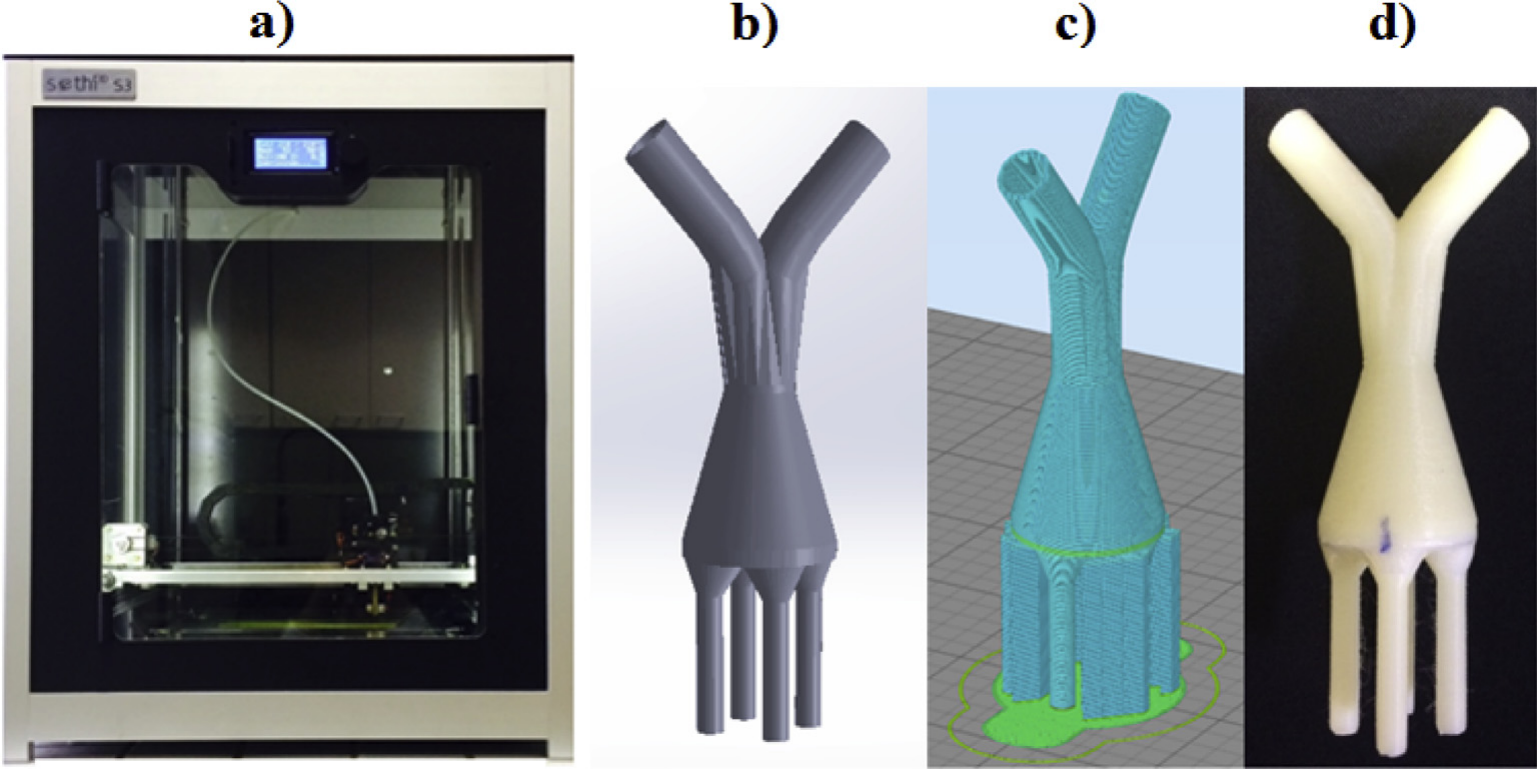
3D printing process: a) Sethi3D S3 printer; b) digital model of the flow distributor; c) slicing on Simplify3D software and d) distributor manufactured on Sethi3D S3 printer.

The 3D printing process were performed using the Sethi3D S3 printer (Fig. 1a) (Sethi3D, Campinas, SP, Brazil). Firstly, the slicing of the digital 3D distributor design model (Fig. 1b) into hundreds or thousands of horizontal layers was carried out using the Simplify3D software (Fig. 1c). The distributor printing process occurred with ABS polymer (Acrylonitrile-Butadiene-Styrene) heated at 235 °C (polymer melt temperature) and by an extruder. The polymer was deposited layer by layer until complete the device confection (Fig. 1d).

The flow distributors were developed using SketchUp software (3D design) based on Zhang et al. [4] and Gomes et al. [5]. Three different flow distributors were proposed in this study: rectangular distributor (RD), conical distributor with obstacle (CDO) and the conical distributor without obstacle (CD). A total of nine flow distributors were developed with 2 central inlets with 6.79 mm of diameter (D_1_) and 4 outlets with 3.10 mm of diameter (D_2_).

**Fig. 2:**
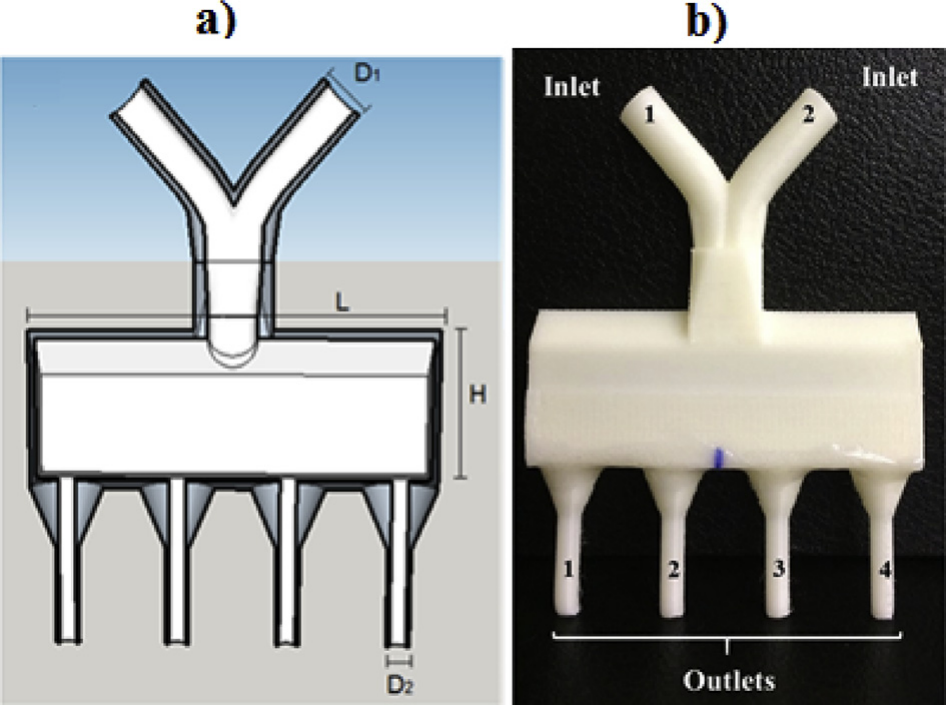
Rectangular distributor (RD) with 26 mm height (RD26): a) digital model with dimensions and b) distributor manufactured by 3D printer.

The rectangular distributor, based on Zhang et al*.* [4], has 72.54 mm of length (L) and 12 mm of width (dimension close to distributor outlet) and was manufactured with 13 mm (RD13), 26 mm (RD26) and 52 mm (RD52) of height (H) (Fig. 2). The G-code of the RD26, which is the file containing all print information, can be downloaded in Supporting Information File 1 at no cost.

**Fig. 3:**
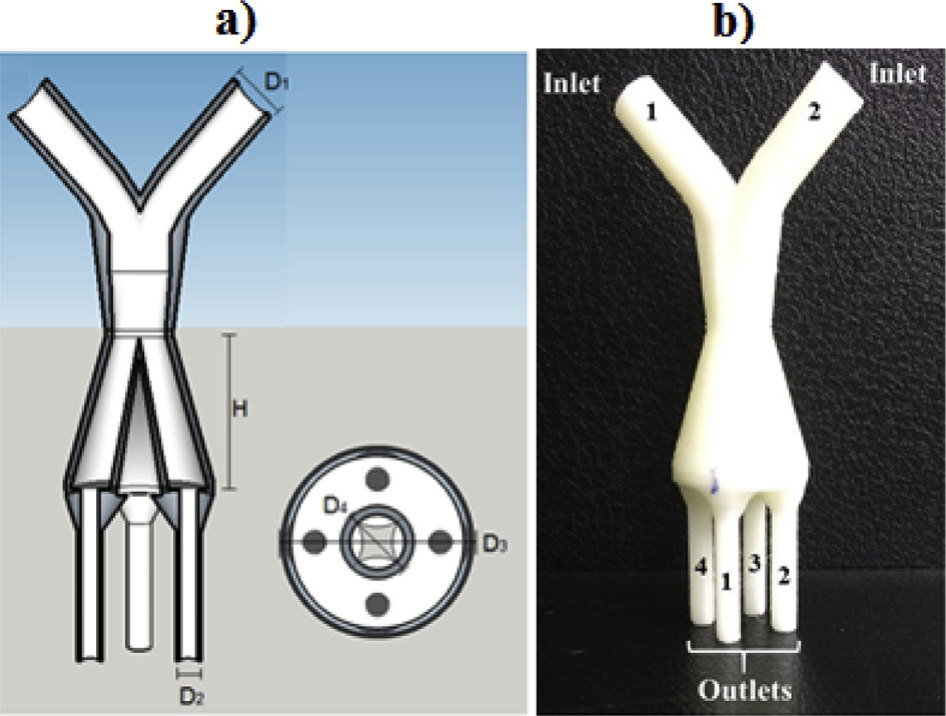
Conical distributor with obstacle with 26 mm height (CDO26): a) digital model with dimensions and b) distributor manufactured by 3D printer.

**Fig. 4:**
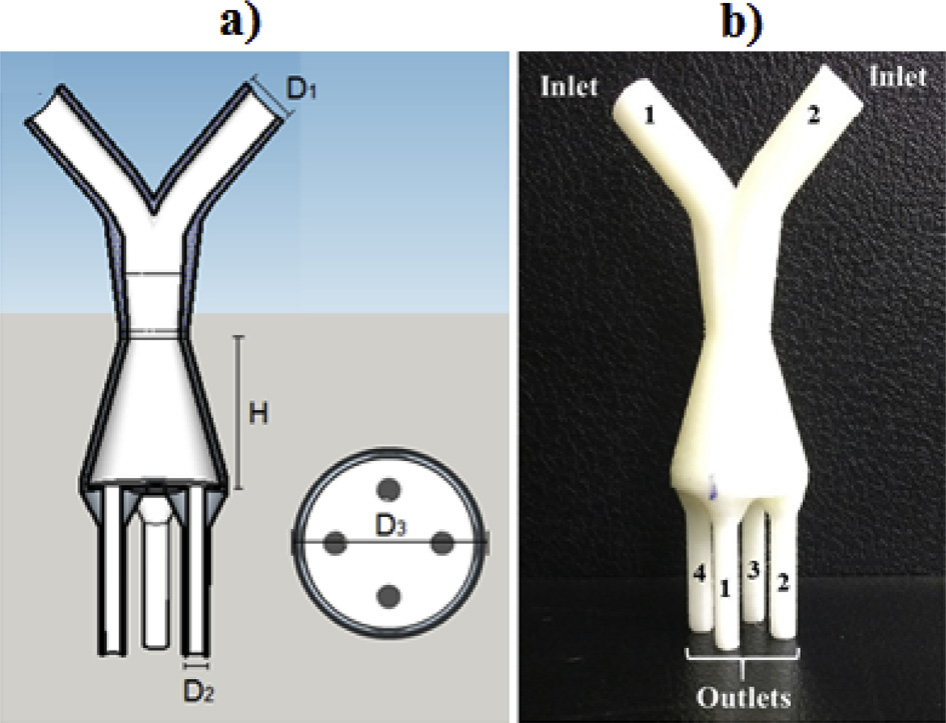
Conical distributor without obstacle with 26 mm height (CD26): a) digital model with dimensions and b) distributor manufactured by 3D printer.

Conical distributors were developed based on Gomes et al. [5] (Fig. 3, Fig. 4). The CDO has a base diameter of 23.56 mm (D_3_) and an obstacle diameter of 10.08 mm (D_4_) (Fig. 3a). The CD has the same CDO dimensions of base diameter (D_3_) (Fig. 4a). Distributors were developed with heights (H) of 13 mm, 26 mm and 52 mm, (Fig. 3, Fig. 4b), and have been named as CDO13, CDO26 and CDO52 for conical distributor with obstacle and CD13, CD26 and CD52 for conical distributor without obstacle.

Table 3 presents 3D printing data of all printed distributors, including the cost of manufacture. Low cost manufacture was verified, once the printing costs were always below US$ 1.14.

**Table 3:** 3D printing data of distributors.

Distributor	3D printing data			
	Height (mm)	Printing time (min)	ABS mass (g)	ABS cost (US$)
RD	13	56	20.42	0.79
	26	68	24.10	0.93
	52	87	29.62	1.14
CDO	13	32	9.08	0.35
	26	37	10.48	0.40
	52	44	12.74	0.49
CD	13	32	9.19	0.35
	26	34	9.91	0.38
	52	46	13.90	0.54

### 2.2 Experimental test of flow distributors

**Fig. 5:**
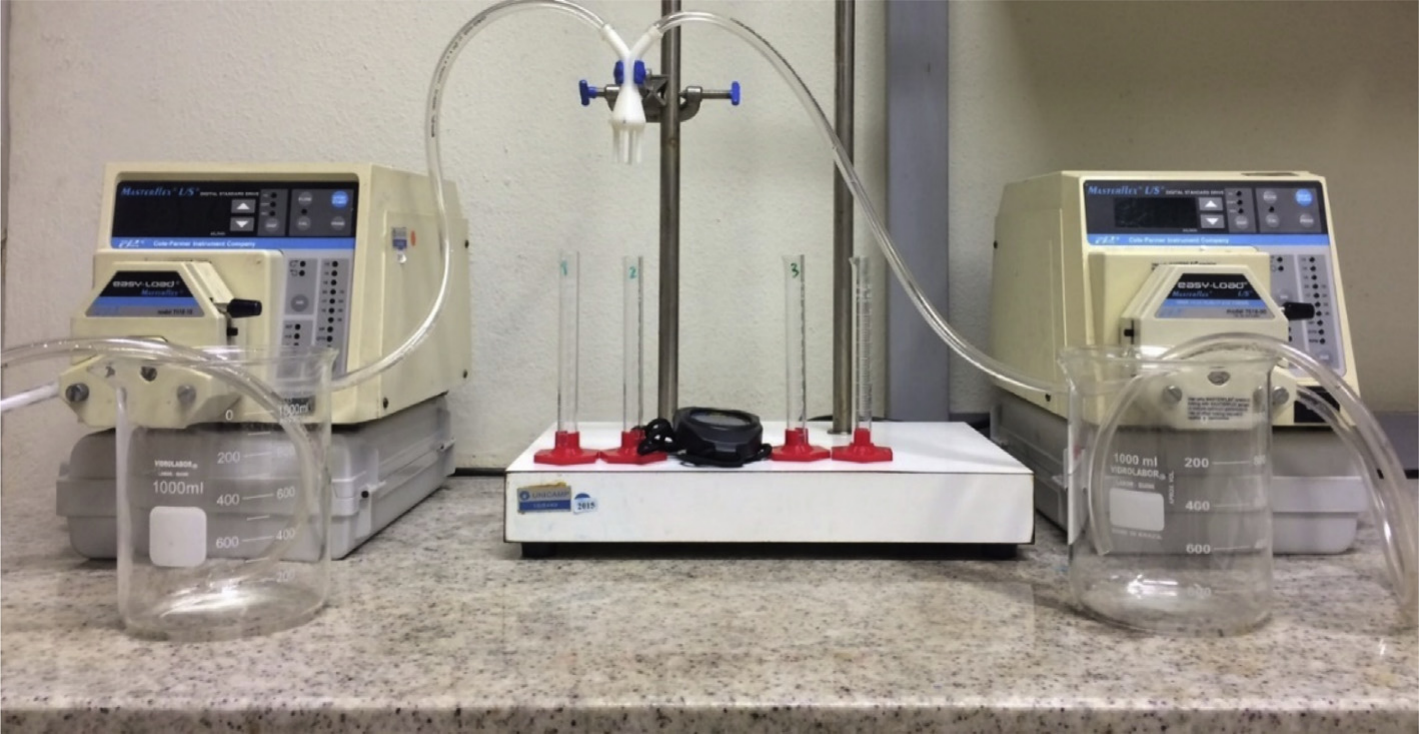
Experimental apparatus for flow distribution test.

Experimental flow tests using tap water were performed to evaluate the flow uniformity. The 3D printed distributors were connected by Masterflex^®^ (Tygon Lab - L/S 17) flexible pipes to 1L beakers containing water. Two Masterflex^®^ L/S^®^ peristaltic pumps were employed to provide the required flow (50, 100, 120, 150, 170 and 200 mL min^−1^). The apparatus arrangement is shown in Fig. 5. The flow measurements at distributor outlets were done in triplicates using graduate test tubes and chronometers, in order to minimize the uncertainties of experimental analyses.
