# Supplementary material for: Flow uniformity data on 3D printed flow distributors
Source: Data Brief. 2019 Feb 28;23:103799. doi: 10.1016/j.dib.2019.103799 (PMC6660602; doi:10.1016/j.dib.2019.103799)
Supplement: Multimedia component 1 [file mmc1.docx]

**Declaration of interests**

The authors declare that they have no known competing financial interests or personal relationships that could have appeared to influence the work reported in this paper.

The authors declare the following financial interests/personal relationships which may be considered as potential competing interests:

The authors declare the following potential competing financial interests - our team filed a patent covering the technology described in this manuscript concerning the conical distributors, millireactors and support structure presented in the present research: "SISTEMA DE ESCALONAMENTO DE PROCESSOS QUÍMICOS, DISTRIBUIDOR CÔNICO DE FLUIDO, ESTRUTURA DE SUPORTE DE MILIRREATORES E MILIRREATOR"; Inventors: H.S. SANTANA, M.G.M. LOPES, V.F. ANDOLPHATO, F.N. RUSSO, J.L. SILVA Jr., M.V.R. ESTEVOM, O.P. TARANTO; Patent number: BR1020180090437, filed on 05/04/2018.
